# Characterization of different stages of corneal infections using anterior segment OCT to enhance monitoring of progression and response to therapy

**DOI:** 10.1038/s41433-025-03952-6

**Published:** 2025-08-20

**Authors:** Farida Omar ElZawahry, Burcin Kepez Yildiz, Frederick Beer, Dalia Said, Harminder S. Dua

**Affiliations:** 1 Division of Clinical Neuroscience, Department of Ophthalmology, University of Nottingham, Nottingham, UK; 2 National Institute of Laser enhanced Sciences, Cairo University, Cairo, Egypt; 3 Başakşehir Çam and Sakura City Hospital, Istanbul, Turkey; 4 Queen Alexandra hospital, Cosham, Portsmouth, UK

**Keywords:** Corneal diseases, Tomography

Infectious Keratitis (IK) is inflammation of the cornea as a result of infection or injury, followed by infection. With early treatment, it is usually curable but if untreated can lead to severe complications and possibly loss of eye [1]. The positive predictive value of physician’s clinical experience had been shown to be very variable depending on the aetiology of the infection. The rate of positive culture in cases of infectious keratitis has varied widely between 40 and 70% due to a variety of limiting factors. For these reasons, delays in diagnosis and treatment are common, and there is a strong necessity to utilise new, reliable modalities of investigation in the diagnosis of IK [2]. Anterior Segment Optical Coherence Tomography (AS-OCT) has become an important adjunctive tool in the diagnosis, evaluation and management of many corneal and anterior segment diseases, allowing a detailed evaluation in a non-contact and safe manner [3].

We report the changes seen on AS-OCT in IK of different aetiologies with a view to establish features in the acute, healing and healed stages. AS-OCT of 45 patients with IK was retrospectively reviewed. AS-OCT scans were performed in the acute stage and at two follow-up time points determined by the progression/resolution of the lesion, aiming to capture the images at the acute stage, mid (resolving) stage and the healed stage. Type of infection, depth and width of infiltrate, stromal vessels, central corneal thickness and each corneal layer were assessed separately at the three mentioned time points. (Table 1).

**Table 1:** Shows the clinical signs detected by OCT.

Central corneal thickness^a^	Epithelial changes	Stromal changes	Oedema:	Descemet’s membrane changes	Corneal blood vessels	Hypopyon and/or inflammatory plaque^b^
	Epithelial bullae	Hyper-reflective lesion in the superficial or deep stroma or both	Mild	Thickness		
	Epithelial irregularity	Hypo-reflective (fluid filled) spaces in the stroma	Moderate	Keratic precipitates		
	Epithelial thickening		Severe	Undulation		
	Epithelial thinning			Detachment^c^ Type 1 / Type 2		
	Epithelial loss					

^a^Measured at the centre of the cornea from epithelium to endothelium.

^b^Infiltrate depth and maximum width were taken measured with the calliper tool of the imaging system, where possible.

^c^Type 1: Detachment of Descemet membrane (DM) and pre Descemet layer. Type 2: Detachment of DM only.

The anterior segment module of the HRT II (Heidelberg retina tomogram, Heidelberg Engineering GmbH, Heidelberg, Germany) (HEYEX I) software was used in scotopic conditions. Forty-two scans of 278 microns intervals, and width of 20° x 10° (11.1 mm × 5.6 mm) to cover the area of infection, were captured.

All patients were treated in accordance with our standard protocols for viral, bacterial, fungal, parasitic (acanthamoeba) and mixed infections. All forty two scans were examined individually for each patient and correlated with the slit lamp examination and images using (Topcon slit lamp SL-D701, Tokyo, Japan). Statistical analysis was carried out using the SPSS® V20 software.

**Fig. 1:**
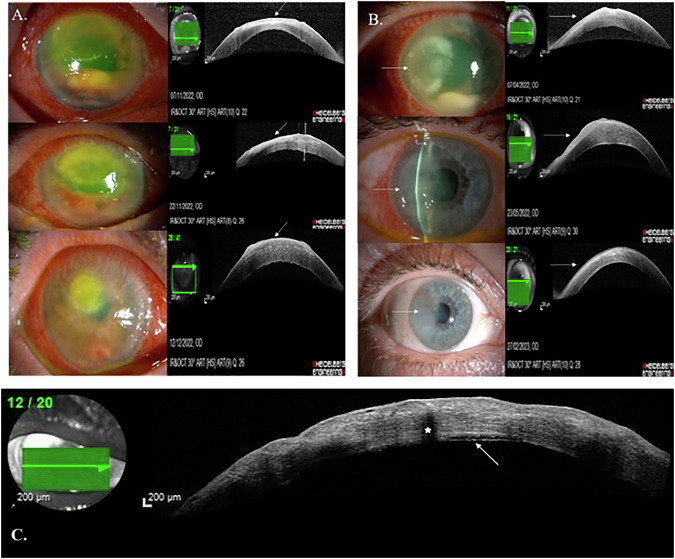
Stages of  Keratitis of different etiologies with Imaging Correlates. **A** Images of different phases of fungal keratitis. Top left, slit lamp diffuse illumination image of the right cornea showing a paracentral active infiltrate with limbal injection and anterior chamber organised hypopyon. Top right, corresponding anterior segment optical coherence tomogram (ASOCT) of the cornea showing an epithelial defect with an underlying hyper-reflective infiltrate involving the superficial-mid stroma (arrow). Middle left, slit lamp diffuse illumination image of the right cornea showing a paracentral healing infiltrate and resolving anterior chamber coagulum. Middle right, corresponding ASOCT of the cornea showing nasal epithelial healing and a decrease in infiltrate thickness and width (arrow). Bottom left, slit lamp diffuse illumination image of the right cornea showing healed epithelium and resolved infiltrate with superficial and deep stromal vascularization. The anterior chamber coagulum has resolved. Bottom right, corresponding ASOCT of the cornea showing raised healed epithelium with no visible underlying hyper-reflectivity (arrow). **B** Images of different phases of bacterial keratitis (Pseudomonas aeruginosa). Top left, slit lamp diffuse illumination image of the right cornea showing a temporal ring-shaped ulcer (arrow) with corneal oedema, hypopyon and severe injection of limbal and conjunctival vessels. Top right, Corresponding ASOCT of the cornea showing an area of epithelial defect overlying a superficial dense hyper-reflective area corresponding to the ulcer (arrow). Middle left, slit lamp slit beam image of the right cornea showing surface irregularity after starting treatment. Middle right, corresponding ASOCT image of the cornea showing thickening and bulging of the superficial stromal tissue associated with a decrease in tissue hyper-reflectivity (arrow). Bottom left, slit lamp diffuse illumination image of the right cornea showing a healed ulcer with no visible corneal oedema. Bottom right, ASOCT of the cornea showing healed epithelium and a visible demarcation line of scar tissue (arrow). **C** Anterior segment optical coherence tomogram of the cornea from a case of fungal keratitis showing a type 1 Descemet membrane detachment (detachment of the pre-Descemet layer and Descemet membrane), which appears as a straight line with inflammatory debris in the space between the detachment and posterior cornea (arrow). Back shadow cast by a blood vessel with active circulation (*).

Viral keratitis (12, 26.7%) was the commonest. Infiltrate and oedema showed thickening but a significant reduction was noticed in the infiltrate thickness during healing. The central corneal thickness showed significant reduction between the acute and healed stage (*p* 0.001) Epithelial defect was evident in all eyes at initial presentation but only 3 (6.7%) had persistent epithelial defect at healed stage with thick epithelium at the margins. Active vessels and infiltrates produced back-shadows that faded as the IK settled [4]. The pre-Descmet layer (PDL) was detached, together with the DM (type 1 DM detachment) in two cases of fungal keratitis (Fig. 1)[5]. All parameters assessed on OCT are reported in Table 1. The study establishes that the reducing thickness of the unaffected (central cornea) and the infiltrate; reducing intensity of the back-shadow of vessels and infiltrate are indicators of resolving IK, which can be detected before clinical signs are apparent.
